# The role of conduction system pacing in patients with atrial fibrillation

**DOI:** 10.3389/fcvm.2023.1187754

**Published:** 2023-05-25

**Authors:** Nadeev Wijesuriya, Vishal Mehta, Felicity De Vere, Marina Strocchi, Jonathan M. Behar, Steven A. Niederer, Christopher A. Rinaldi

**Affiliations:** ^1^ School of Biomedical Engineering and Imaging Sciences, King’s College London, London, United Kingdom; ^2^ Department of Cardiology, Guy’s and St Thomas’ NHS Foundation Trust, London, United Kingdom; ^3^ Research and Innovation Cluster, Alan Turing Institute, London, United Kingdom

**Keywords:** left bundle area pacing, his bundle pacing, atrial fibrillation, cardiac resynchonization therapy, heart failiure, AV nodal ablation

## Abstract

Conduction system pacing (CSP) has emerged as a promising novel delivery method for Cardiac Resynchronisation Therapy (CRT), providing an alternative to conventional biventricular epicardial (BiV) pacing in indicated patients. Despite increasing popularity and widespread uptake, CSP has rarely been specifically examined in patients with atrial fibrillation (AF), a cohort which forms a significant proportion of the heart failure (HF) population. In this review, we first examine the mechanistic evidence for the importance of sinus rhythm (SR) in CSP by allowing adjustment of atrioventricular delays (AVD) to achieve the optimal electrical response, and thus, whether the efficacy of CSP may be significantly attenuated compared to conventional BiV pacing in the presence of AF. We next evaluate the largest clinical body of evidence in this field, related to patients receiving CSP following atrioventricular nodal ablation (AVNA) for AF. Finally, we discuss how future research may be designed to address the vital question of how effective CSP in AF patients is, and the potential hurdles we may face in delivering such studies.

## Introduction

Cardiac Resynchronisation Therapy (CRT) is a cornerstone in the electrical treatment of heart failure (1). Conventional CRT involves biventricular (BiV) pacing from transvenous leads in the right ventricle (RV) and a coronary sinus branch to provide epicardial left ventricular (LV) stimulation. The widespread uptake of CRT has been driven by evidence showing significant benefits in both hospitalisations and mortality for patients with symptomatic dyssynchronous heart failure, that is, those with a LV ejection fraction (EF) of below 35%, and a QRS duration (QRSd) of greater than 130 milliseconds (ms) on a 12 lead electrocardiograph (ECG) (2). In recent years, the indications for CRT have expanded, with studies showing benefits in patients with moderate LV dysfunction who have a high burden of RV pacing (3) and in those requiring an atrioventricular node ablation (AVNA) (4).

Despite its success, there is a significant proportion of patients who either do not derive clinical benefit from conventional CRT (5), or who cannot be treated due to failure of LV lead implantation or inadequate LV lead performance due to issues such as high thresholds and phrenic nerve stimulation (6). Conduction system pacing (CSP), that is, stimulation of His-Purkinje tissue using a transvenous lead-based system (7), is becoming increasingly popular, not only as a “bail-out” treatment in this population, but also potentially as a first-line option in selected patients (8). Initial studies on CSP focused on His-Bundle pacing (HBP), built on the attractive concept of restoring completely physiological ventricular activation. HBP achieves excellent cardiac resynchronisation, but implantation can be difficult, with success rates varying from 56%–95% (7, 9, 10). Challenges such as ventricular under-sensing, rising thresholds and requirement for lead revisions have also been observed during long-term follow up (11, 12). Left Bundle Branch Pacing (LBBP) is a relatively novel form of CSP which involves screwing a pacing lead deep into the interventricular septum from the RV in order to capture the left bundle system (13, 14). This technique has shown encouraging results from observational studies, with reported success rates of 80%–94% and significant improvements in LV function (13, 15). Robust data from randomized trials, however, is currently lacking, and despite widespread uptake and investigation of CSP, important questions on its use remain.

An area of significant clinical importance is the role of CSP both in the presence of, and in the treatment of atrial fibrillation (AF). AF is the most prevalent arrhythmia worldwide (16), and is very common in the heart failure population, affecting up to a third of patients receiving CRT (17). Several studies have reported an attenuation of clinical benefit achieved with CRT in the presence of AF (18–20). There are likely several mechanisms behind this, including the reduction in cardiac output associated with loss of atrial systole (21), low BiV pacing percentage due to uncontrolled ventricular rates (22), and increased risk of inappropriate shocks from implantable cardioverter defibrillators (ICDs) (23).

In view of the significant deleterious consequences that AF has on conventional CRT, and the increasing use of CSP in this patient population, it is crucial to examine whether the presence of AF affects outcomes of HBP and LBBP. This article focuses on two areas. First, we evaluate the possible impact of AF on CSP, specifically LBBP, due to the inability to improve ventricular dyssynchrony by adjusting AVDs to achieve fusion pacing with intrinsic RV conduction (13). Next we review the largest clinical body of evidence in the case of CSP use following AVNA for AF. Finally, we discuss how future studies may address the important issue of CSP efficacy in patients with AF.

## Is an intact sinus rhythm important in conduction system pacing?

The primary attraction of CSP is theoretically in more closely mimicking physiological ventricular electrical activation, thus minimising ventricular dyssynchrony. There is emerging evidence that the presence of sinus rhythm (SR) and intact AV node conduction may be crucial in this process. This raises the question of CSP efficacy, particularly LBBP efficacy, when patients are in AF or have complete heart block (CHB), which is highly pertinent in the AVNA population. The majority of evidence in this area is in the form of in-silico models, mechanistic and observational studies.

Curila et al. examined the activation patterns of patients receiving LBBP in a series of studies utilising ultra-high frequency ECG during temporary pacing protocols (24, 25). These studies demonstrated that LBB capture, whilst leading to accelerated LV lateral wall activation, led to increased interventricular dyssynchrony compared to LV septal myocardial pacing (LVSMP) due to late RV activation. Interestingly, this dyssynchrony was reduced in a proportion patients where bipolar anodal septal capture could be achieved, leading to simultaneous RBB, LV septal and LBB capture, compared to unipolar LBB capture in the same patients (26).

**Figure 1:**
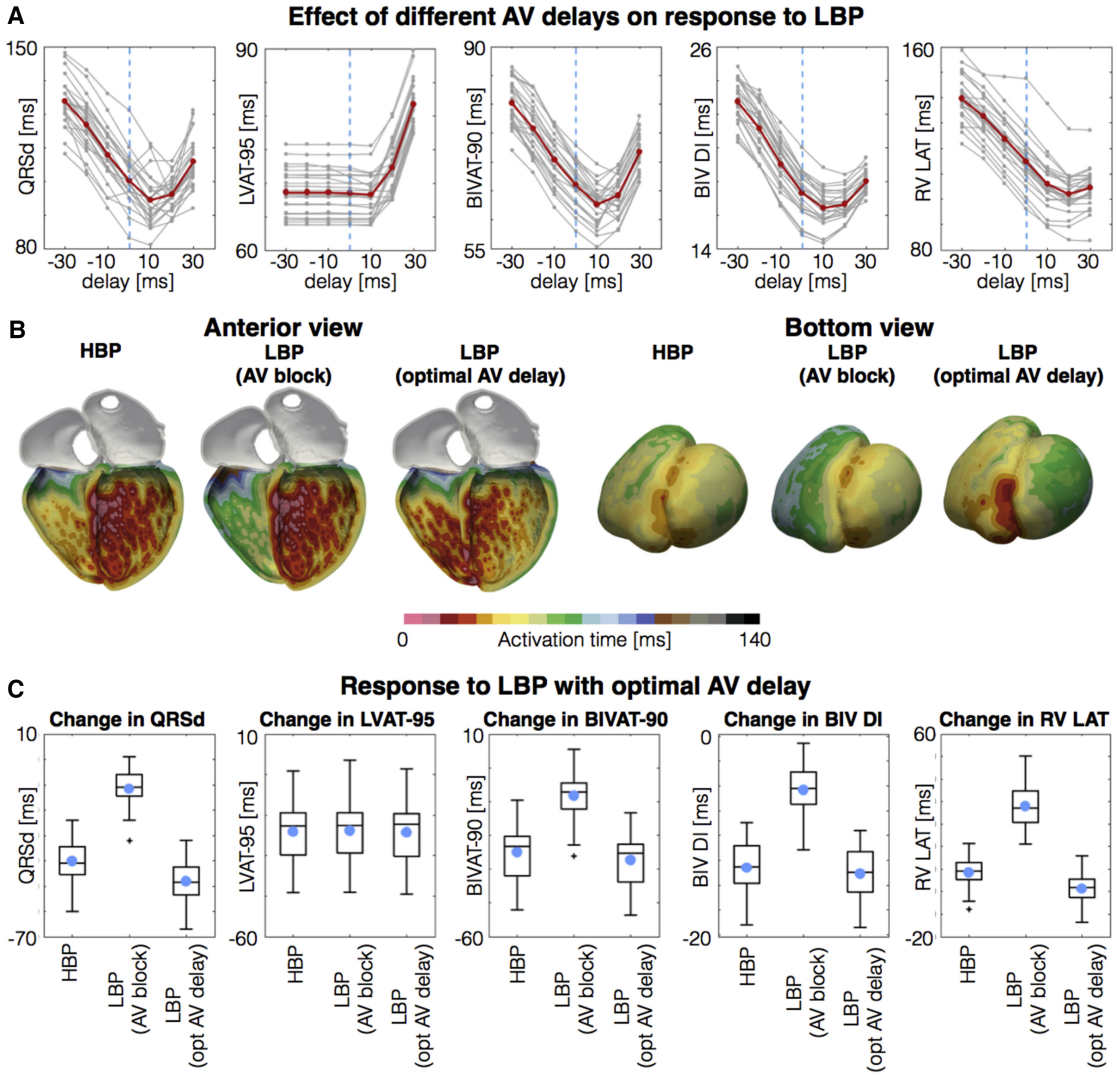
Response to LBP with optimized AV delay. (**A**) Response measures simulated with different AV delays. Negative and postive delays mean that the left bundle is paced before and after the atrial stimulus enters the His, respectively. *Gray lines* represent the patients. *Red lines* represent the mean. (**B**) Activation times with selective HBP, selective LBP with complete AV block and with optimal AV delay. (**C**) Boxplots of change in QRSd, LVAT-95, BIVAT-90, BIV DI, and RV Latest AT for selective HBP, selective LBP with complete AV block and with optimal AV delay. *Light blue circles* represent mean values. *Plus symbols* represent outliers. AV, atrioventricular; BIV DI, biventricular dyssynchronous index; BIVAT-90, 90% biventricular activation time; HBP, his-bundle pacing; LBP, left bundle pacing; LVAT-95, 95% left ventricular activation time; QRSd, QRS duration; RV LAT, right ventricular latest activation time. Reproduced from Strocchi et al (27) with permission.

Strocchi et al. (27) simulated BiV activation on 24 four-chamber heart meshes in the presence of left bundle branch block (LBBB). The authors simulated conventional BiV epicardial pacing, BiV endocardial pacing with LV lead at the lateral wall, BiV endocardial pacing with LV lead at the LV septum, HBP, and LBBP. They found that both selective and non-selective HBP improved response metrics such as QRSd, LV activation time (LVAT), RV activation time (RVAT), BIV activation time (BiVAT) and BiV dyssynchrony index (BiV-DI) compared to BiV epicardial or endocardial pacing. With regards to LBBP, they reported that in the presence of simulated CHB, LBBP led to increased ventricular dyssynchrony compared to HBP due to increases in RVAT (141.3 ± 10.0 ms versus 111.8 ± 10.4 ms). RVAT, and therefore BiVAT, was improved in patients with intact native conduction when AV delay (AVD) optimisation was performed to allow intrinsic RV activation via the right bundle branch (RBB), with response metrics similar to those achieved with HBP (Figure 1). In the presence of CHB, RV apical pacing was required in addition to LV LBBP in order to maintain BiV synchrony by preventing late RV activation.

**Figure 2:**
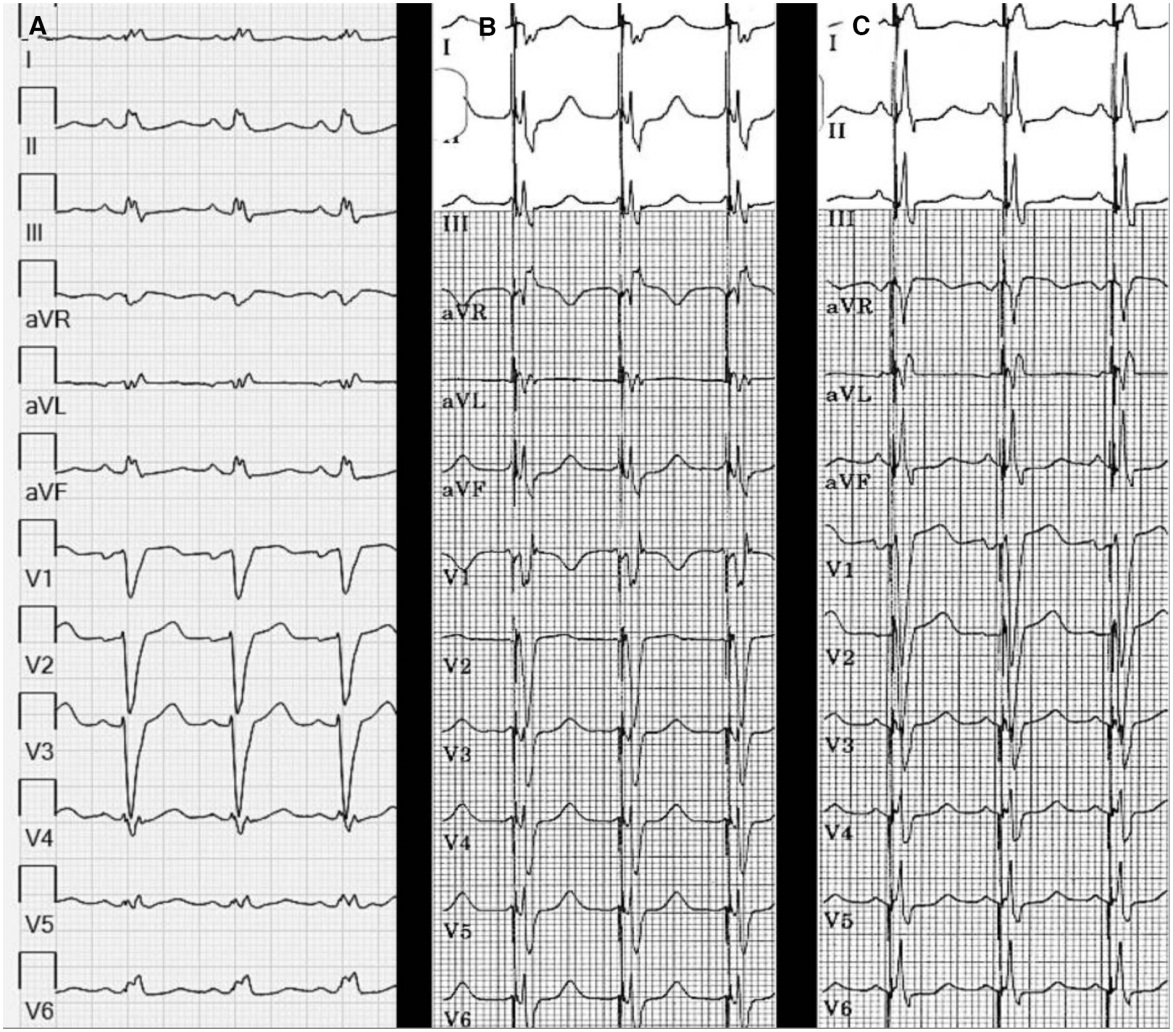
Response to LBBP. (**A**) ECG showing SR and LBBB with QRSd 156 ms. (**B**) Pacing with AV delay set at 40 ms resulted in resulted in QRSd of 128 ms. (**C**) Pacing with AV delay set at 80 ms resulted in normalization of QRSd to 120 ms.

This phenomenon has also been observed in-vivo. Padala et al. (15) report a case of LBBP as a bailout treatment for a patient with ischaemic cardiomyopathy and several failed coronary sinus lead implants. The baseline ECG showed sinus rhythm and LBBB with QRSd 156 ms. Pacing with AVD set at 40 ms resulted in LBBA capture with RBBB pattern in lead V1 with QRSd of 128 ms. Pacing with AVD set at 80 ms resulted in normalization of QRSd to 120 ms, due to fusion between anterograde RBBB conduction and LBBP (Figure 2).

These results were supported by Huang et al. (28), who reported the outcomes of 63 patients with non-ischaemic cardiomyopathy and LBBB implanted with a LBBP device. They found that during unipolar tip LBBP, the QRSd narrowed significantly from 169 ± 16 ms at baseline to 118 ± 12 ms, and then further to 103 ± 9 ms (*p* < 0.05) during LBBP fused with native conduction by optimizing AVD.

A study performed by Wu et al. (29) again demonstrated the possible benefits of AVD optimisation. In a non-randomised comparison, 49 patients received HBP, 32 received LBBP and 54 received standard BiV pacing. In this study, HBP resulted in normalization of QRS morphology, with the mean paced QRSd of 100.7 ± 15.3 ms; LBBP resulted in a paced QRS morphology of RBBB pattern, with a mean paced QRSd was 110.8 ± 11.1 ms; the QRSd further reduced to 104.3 ± 8.1 ms with AVD optimization to promote fusion pacing. Maximum reduction in QRS duration was observed during HBP (Δ QRSd = 69 ms) and fused LBBP (Δ QRSd = 65 ms). A modest improvement was seen with LBBP in the absence of AVD optimisation (Δ QRS duration = 56 ms), and minor improvements were seen with BiV pacing (Δ QRS duration = 26 ms).

These studies suggest that, in order to achieve similar electrical outcomes to HBP, LBBP needs to be combined with AVD optimisation to obtain fusion with native RBBB conduction. However, the functional benefit gained from a relatively small improvement in QRSd remains unclear. In an acute electrocardiographic imaging (ECGi) and haemodynamic study using temporary pacing protocols, Ali et al. (30) found that the optimal AVD to achieve fusion pacing with LBBP resulted in the best acute systolic blood pressure increase in only 6 out of 19 patients in the study. This indicated that the relationship between the AVD and LBBP is more complex than simply achieving ventricular resynchronisation. Contributory factors such as the duration of passive ventricular filling, atrial mechanical systole and RV function mean that the optimal AVD for an individual patient is not a metric which can be determined purely electrically. Indeed, studies have shown that in conventional CRT, the optimal AVD for filling is different to the optimal AVD for fusion (31).

In addition, no in-vivo studies have thus far demonstrated that AVD optimisation to achieve fusion impacts the acute haemodynamic performance of LBBP when compared to conventional BiV epicardial pacing. For example, in an acute ECGi and haemodynamic study, Elliott et al. (32) reported that LBBP, HBP and BiV endocardial pacing all outperform conventional BiV epicardial pacing in the absence of AVD optimisation. As such, the presence or absence of AF may not have a significant enough bearing on the efficacy of LBBP to change treatment decisions surrounding CRT modality.

There is some comparative clinical evidence to support this. Huang et al. (28) reported an observational study of patients with non-ischaemic cardiomyopathy and LVEF < 50% who received LBBP. At 6-months follow up, there was no significant difference in improvement in LVEF or New York Heart Association (NYHA) functional class between the patients in SR (*n* = 48) and those in persistent AF (*n* = 14). This could suggest that improved ventricular synchrony due to less delayed RV activation during AVD optimisation (or indeed, anodal capture in applicable instances) may not necessarily translate to an improvement in clinical outcomes, and that examination of larger studies reporting longer term data will be needed to form more robust conclusions.

### Conduction system pacing in patients receiving atrioventricular node ablation

AVNA has been shown to improve CRT outcomes in patients with AF (18). Gasparini et al. reported results from the CERTIFY study (33), a multinational registry comprising 1,338 patients with AF and 6,046 patients in SR. They found that at median follow-up of 37 months, total mortality rates in patients with AF treated with AVNA (*n* = 443) were similar to patients in SR. In contrast, patients with AF treated with medications alone had a significantly higher mortality rate than patients in SR. The authors postulated that this was primarily due to an increase in BiV pacing percentage in the AVNA group compared to the non-AVNA group (96% vs. 87%).

With regards to the benefits of AVNA plus CRT over pharmacological therapy in patients with a narrow native QRS and symptomatic permanent AF, the APAF-CRT study (4) reported all-cause mortality in 133 patients randomised to pharmacological therapy or AVNA plus conventional BiV CRT. There was a significant reduction in all-cause mortality in the AVNA group (11% vs. 29%, *p* = 0.004), with similar results seen in both patients whose LVEF was <35% or >35%. Previous studies have demonstrated that CRT outperforms RV pacing alone in patients receiving AVNA (34). Evidence such as this has motivated the examination of CSP in patients requiring AVNA. This is currently where the bulk of clinical evidence is derived on the use of CSP in the context of AF.

Several small, retrospective studies have demonstrated the feasibility of HBP and AVNA. Vijayaraman et al. (35) reported the results of 42 consecutive patients who underwent HBP and AVNA, with an overall procedural success rate of 95%. They documented that the successful AVNA site was at or below the ring electrode in 22 patients (no acute change in HBP threshold); above the ring electrode in 13 patients, and from the left side in 2 patients (acute increase in HBP threshold in 7 of 15 patients). LVEF increased from 43% ± 13% to 50% ± 11% (*p* = 0.01), and NYHA functional status improved from 2.5 ± 0.5 to 1.9 ± 0.5 (*p* = 0.04). The overall increase in HBP threshold was 0.6 V at a mean follow up of 19 months. One patient required HBP lead revision. The authors concluded that HBP was a feasible technique for delivery of CRT post-AVNA. The study does, however, highlight the limitations of this intervention with regards to long-term lead performance, which may be ablation-site dependent. Similar success rates and echocardiographic improvements were reported in a retrospective study of 94 patients performed by Su et al. (36), as well as in several smaller studies (37, 38).

In terms of potential benefits of CSP and AVNA approach compared to pharmacological rate control therapy, Wang et al. (39) retrospectively analysed data from 86 non pacing-dependent patients with persistent AF undergoing ICD implantation. Fifty-two patients also underwent CSP to achieve CRT (HBP *n* = 44; LBBP *n* = 8), followed by an AVNA, while the remaining patients received pharmacological rate control. They reported that patients receiving CSP had lower incidence of inappropriate shock (15.6% vs. 0%, *p* < 0.01), and demonstrated an improvement in LVEF (15% vs. 3%, *p* < 0.001) compared to patients receiving ICD implantation and pharmacological rate control. Whilst this evidence is not as robust as the APAF-CRT prospective randomised trial (4), it does signal that CSP may perform similarly to conventional CRT post-AVNA with regards to added benefit over pharmacological rate control.

Data comparing CSP to conventional BiV pacing post-AVNA has also been published. In a small, retrospective analysis of 24 patients (HBP *n* = 12, BiV CRT *n* = 12), Zizek et al. (40) reported improved echo outcomes in the HBP group. These results have been subsequently supported in the ALTERNATIVE-AF trial (41). This was a randomized crossover trial which recruited patients with persistent AF and a LVEF ≤ 40%. All patients underwent AVNA and received both HBP and conventional BIV pacing. Fifty patients were randomized to either HBP or BiV pacing for 9 months (phase 1), then were switched to the alternative pacing modality for the next 9 months (phase 2), with 38 patients completing both phases, thus being included in the cross-over analysis. The primary endpoint was change in LVEF. A significant improvement in LVEF was observed with HBP compared to BiV pacing (phase 1: ΔLVEF_HBP_ 21.3% and ΔLVEF_BiV_ 16.7%; phase 2: ΔLVEF_HBP_ 3.5% and ΔLVEF_BiV_ −2.4%; p_generalized additive model_ = 0.015). Significant improvements in LV end-diastolic diameter, NYHA functional class, and B-type natriuretic peptide level were observed with both pacing modalities compared with baseline, whereas no significant differences were observed between HBP and BiV pacing.

Vijayaraman et al. (42) published a non-randomised on-treatment comparison of 223 patients, with 110 having received CSP (84 HBP, 26 LBBP), and 113 receiving conventional pacing (CP, RVP or BiV pacing based on operator discretion). QRSd increased from 103 ± 30 ms to 124 ± 20 ms (*p* < .01) in CSP and 119 ± 32 ms to 162 ± 24 ms in CP (*p* < .001). During a mean follow-up of 27 ± 19 months, LVEF significantly increased from 46.5% ± 14.2% to 51.9% ± 11.2% (p = .02) in CSP and 36.4% ± 16.1% to 39.5% ± 16% (*p* = .04) in CP. The primary combined endpoint of time to death or heart failure hospitalisation was significantly reduced in CSP compared to CP (48% vs. 62%; hazard ratio 0.61, 95% CI: 0.42–0.89, *p* < .01). Of note, whilst this was a large study, 24% of patients with impaired LVEF received RVP, which likely negatively impacted outcomes in the CP cohort.

As yet, there are no randomised control trials examining the role of LBBP following AVNA, however, retrospective analyses have been performed. Cai et al. (43) prospectively enrolled 99 patients who received AVNA and LBBP for AF rate control. Implant success rate was 100%. LVEF improved from baseline 30.3% ± 4.9 to 47.3% ± 14.5 at 12 months follow-up in HF patients with reduced LVEF and from baseline 56.3% ± 12.1 to 62.3% ± 9.1 in HF patients with preserved LVEF (both *p* < 0.001). Outcomes of 86 of these patients were compared to a propensity-matched cohort who had received HBP. No significant differences in echocardiographic or clinical outcomes were observed between HBP and LBBP, however, lower thresholds, greater sensed R-wave amplitudes, and fewer complications were observed in the propensity-matched LBBP group (*p* < 0.05).

Pillai et al. (44) performed a retrospective analysis directly comparing 98 consecutive patients referred for CSP leads over a 7 year period, where 48 received HBP and 50 received LBBP prior to AVNA. The authors reported an acute success rate of the AVNA procedure of 94% vs. 100% (*p* = .11) in HBP vs. LBBP groups. Seven (14%) redo AVNA procedures were required in the HBP group. Mean procedural time (44 ± 24 min vs. 34 ± 16 min; *p* = .02) and mean fluoroscopy time (16 ± 18 min vs. 7 ± 6 min; *p* < .001) were significantly longer in the HBP vs. LBBP group. An acute rise in threshold was observed in 8 HBP cases (14.5%), and 4 (8%) developed exit block after AVNA. Chronic HBP threshold ≥2.5 V was seen in 23 patients (48%), and 4 (8%) of HBP leads were deactivated. Both forms of CSP preserved LVEF post-AVNA in patients with a baseline LVEF > 50%, and significantly improved function in those with a baseline LVEF <50%. The authors concluded that whilst CSP with either HBP or LBBP would preserve or restore LV systolic function post-AVNA, fewer acute procedural complications during AVNA and fewer long-term lead-related performance issues were observed in the LBBP group.

In terms of future directions, there have been both case reports (45) and series (46) demonstrating the feasibility of simultaneous CSP and AVNA. Vijayaraman et al. (46) reported procedural results of a cohort receiving HBP at the same time as AVNA. In 22 of 25 patients, AVNA was feasible via axillary access, with femoral access only required in 3 patients early in the centre's experience. The authors suggest that this combined procedure was not only safe and effective, but may reduce procedural duration and allow for early ambulation.

Taken together, the early evidence suggests that CSP may be a viable alternative to conventional BiV pacing post-AVNA for AF. The technical issues presented by the proximity of ablation site to HBP leads, and long-term issues with HBP lead performance mean that LBBP may be more beneficial moving forwards in this field. More robust data from randomised trials comparing LBBP with BiV pacing post-AVNA will be important in this regard.

## Discussion

Determining the efficacy of CSP in patients with AF is a crucial area to evaluate, given the increasingly widespread use of CSP, and the high burden of AF in the eligible populations.

A review of in-silico and mechanistic in-vivo evidence would suggest that HBP may provide improved electrical synchrony compared to conventional epicardial BiV pacing. However, with HBP potentially limited by technical difficulties at implantation and long-term lead performance issues, examining how LBBP improves dyssynchrony is perhaps more relevant. The current data does suggest that the presence of intact SR, and thus the ability to adjust paced AVDs to achieve fusion pacing with intrinsic RV conduction, does improve LBBP acute electrical outcomes. The concept of fusion pacing and AVD adjustment is not a novel concept, and has in fact been the subject of interest in conventional CRT (47). However, despite studies suggesting that the primary benefit in CRT and CSP may be related to AVD shortening (48), this theory has not been borne out in larger trials of AVD optimisation in CRT (49), perhaps due to the complex relationship between the AVD and cardiac output, with acute ventricular resynchronisation being only a part of the whole story. With regards to LBBP, it is yet to be determined whether AVD optimisation leads to improved long-term outcomes. Randomised trials comparing LBBP in SR and AF populations may address this, but given the modest improvements observed in acute electrical outcomes, prohibitively large sample sizes may be required to detect small differences in hard clinical endpoints. Of note, clinical effect sizes may be further diluted by a proportion of patients who exhibit anodal septal capture (26, 50). Such patients may not gain electrical benefit from AVD optimisation, as preservation of intrinsic RBB conduction would theoretically not be necessary in such instances. Another potential avenue of interest is whether therapies such as His-Optimised CRT (HOT-CRT) or Left Bundle Branch Optimised CRT (LOT-CRT) will be beneficial in AF to improve fusion in the absence of AVD optimisation (51, 52). These interventions have shown some promise at improving synchronisation in small observational studies, but their specific benefits in AF patients has as yet not been examined.

Our most substantial clinical body of evidence for CSP in AF comes from the post-AVNA population. These data so far suggest that CSP may be a viable alternative to BiV pacing in this group. However, larger prospective comparative studies are needed to determine whether CSP is equivalent in these cases. Real world data, such as registry, is also very valuable in this field, especially with regards to HBP, where trials performed in high-volume centres by experienced operators may under-estimate the complication rate and with this intervention, which is more technically challenging than LBBP.

Having said this, the most impactful path forward in furthering the field, of course, lies in randomised control trials, ideally studies recruiting exclusively patients with AF comparing CSP vs. BiV pacing in both traditional CRT populations and AVNA populations. There are inherent challenges, however, that come with designing such trials. Firstly, large sample sizes will be needed to adequately power these studies, and this may present problems, especially when the eligible recruitment pool is reduced. Slow recruitment leads to longer duration studies which in turn increases costs as well as drop-out rates. Perhaps equally relevant is that AF patients tend to be under-represented in large clinical CRT trials (53). With the driving force behind the largest studies primarily involving commercial funding and/or sponsorship, industry partners may be less motivated to support trials in AF patients, where the beneficial effects of these devices is likely to be attenuated, and regulatory approvals for broad indications have already been granted on the basis of previous evidence. It is vital, therefore, that well designed investigator-initiated studies are prioritised in the future. In an era where there are a plethora of available CRT treatments, a personalised approach may be needed for individual patients based on a variety of factors, including AF. Large, randomised trials are needed to provide the strong evidence base, crucial in informing the decisions.
